# Supplementary material for: The future(s) of unpaid work: How susceptible do experts from different backgrounds think the domestic sphere is to automation?
Source: PLoS One. 2023 Feb 22;18(2):e0281282. doi: 10.1371/journal.pone.0281282 (PMC9946198; doi:10.1371/journal.pone.0281282)
Supplement: S3 File — (DOCX) [file pone.0281282.s004.docx]

Questionnaire UK version

The structure of the codebook follows the original structure applied in the online survey
including instructions to the respondents. It includes information on all variables available in the dataset: variable name, variable label, coding and values. Each variable is described as follows:

| **Variable label** | **Variable name/question** |
| --- | --- |
| Value label 1 | Value 1 |
| Value label 2 | Value 2 |
| … | … |

**Sampling information**

| **country** | **Country** |
| --- | --- |
| 1 | Japan |
| 2 | UK |

| **respID** | **Respondent ID** |
| --- | --- |

**Instructions**

In the following pages, we will ask you about 17 different types of unpaid housework tasks, such as cooking, dish washing, cleaning, laundry, ironing, and physical childcare. We will also provide you with a brief description of each task, and statistics on how much time UK adults are currently spending on the task (UKTUS 2015).

For each task, we will ask you to predict:

**5 years from now, what percentage of the time that currently goes into this task can be automated? (0-100%)**

**10 years from now, what percentage of the time that currently goes into this task can be automated? (0-100%)**

"Automated" should be understood in a **very wide sense** here as referring to any use of "AI" technology. For instance, it could mean that a person purchases a robot that does some part of the work for them. But it could also mean that the person uses a mobile app that sends a human to do some of the work, as long as that app is made possible by some "AI" technology (e.g. efficient scheduling and routing algorithms; machine learning of customer preferences). Automation could also refer to new smart materials or infrastructures that reduce the amount of time that this task requires. As you consider your answer, please first imagine what kind of an automation solution, if any, might be used.

We are asking how much of the *current* household time use on this task future automation could eliminate. If you answer 0%, it means that you believe that people will have to spend the same amount of time on the task as they are spending today. If you answer 100%, it means that people will not have to spend any time on this task in the future. You can count automation technologies that already exist today but are not widely used, due to e.g. their cost.

We ask you to predict whether automating the task would be ***technically feasible****,*not whether people would actually use such automation. For instance, baking, childcare, and window shopping are examples of tasks that people might not want to automate further even if it was technically feasible. We ask you to ignore this, and only consider the technical feasibility. We are conducting a separate large-scale consumer survey to study consumers' attitudes towards automating different household tasks.

**5 years from now, how much will it cost for a household to use this automation for one year?**

**10 years from now, how much will it cost for a household to use this automation for one year?**

We will also ask you to predict the *cost* of using the automation solution that you have imagined. We realise that this is extremely difficult to predict, as it can depend on numerous factors, such as production costs, demand, competition, marketing, business models (e.g. advertising supported), policy environment (e.g. subsidies to help elderly people live independently), and so on. For this reason, we are only asking you to provide your best guess of the *magnitude* of the price, in terms of the number of zeroes: £0-10, £11-100, £101-1,000 and so on.

For example, you could use your best guess of how such a solution would be marketed: e.g., as a mass-market subscription service (£10/month = £120/year), or as a high-end household appliance (£1,500 purchase price, 3-year replacement cycle = £500/year). Another option is to use your best guess of the magnitude of the production costs and assume that the solution is priced at cost.

Both the technical feasibility and the cost of future automation technologies are extremely difficult to predict using quantitative methods such as statistical generalizations from past data. In such situations, the expert Delphi method has been shown to be capable of producing reasonable ballpark estimates, by aggregating diverse information from many sources.

**You might not always feel that your responses are useful or well-informed, but rest assured that your unique perspective represents a valuable contribution to the overall result.**

The ultimate goal of this research project is to forecast future changes in time spent on unpaid work by gender, age, and income group. We do this by combining empirical data on current time use with the predictions obtained from this Delphi survey, and with consumer technology attitude estimates obtained from our consumer survey.

*In the questionnaire we describe 17 tasks. After each task we ask the experts to predict the degree of automation and the associated cost.*

| **task** | **Task** |
| --- | --- |
| 1 | Cooking |
| 2 | Dish washing |
| 3 | Cleaning |
| 4 | Making clothes |
| 5 | Laundry |
| 6 | Iron |
| 7 | Garden |
| 8 | Pet |
| 9 | House and car maintenance |
| 10 | Grocery shopping |
| 11 | Shopping |
| 12 | Service use |
| 13 | Physical child care |
| 14 | Teaching child |
| 15 | Interacting with child |
| 16 | Escorting child |
| 17 | Care for adult |

*Tasks descriptions:*

**1 Cooking**

Cooking consists of things like choosing, washing, and chopping ingredients; chopping, frying, boiling, and heating food in the oven; setting the table, and serving the food; making coffee, preparing snacks, and baking sweets.

In the UK, 80% of women engage in cooking on an average day, spending on average 60 minutes on this task. Of men, 54% cook, spending 42 minutes on it.

**2** **Dish washing**

Dish washing consists of things like collecting empty dishes after a meal, washing dishes, loading/unloading the dishwasher, and placing dishes back in the cupboard.

In the UK, 46% of women engage in dish washing on an average day, spending on average 29 minutes on this task. Of men, 25% engage in dish washing, spending 24 minutes on it.

**3 Household cleaning**

Cleaning the household consists of things like cleaning floors, making beds, dusting surfaces, arranging household items, cleaning the bathroom, cleaning the kitchen, separating waste, taking out the waste, and watering indoor plants.

In the UK, 55% of women engage in home cleaning on an average day, spending on average 59 minutes on this task. Of men, 31% clean the home, spending 46 minutes on it.

**4 Making and mending clothes**

Making and mending clothes consists of things like knitting, sewing, and using a sewing machine to make and repair clothes, curtains, and accessories.

In the UK, 2% of women engage in making and mending textiles on an average day, spending on average 89 minutes on this task. Of men, less than 1% make and mend textiles, spending 32 minutes on it.

**5 Laundry**

Laundry consists of things like sorting items to be washed, loading and unloading the washing machine, washing clothes and textiles by hand, hanging out items to dry, and putting them away into a wardrobe when they don't need to be ironed.

Note: Ironing and putting items away after ironing is a separate task.

In the UK, 29% of women engage in doing laundry on an average day, spending on average 31 minutes on this task. Of men, 7% do laundry, spending 27 minutes on it.

**6 Ironing and folding**

Ironing and folding consists of things like ironing and mangling clothes and sheets, and folding and putting them into the wardrobe after ironing.

In the UK, 13% of women engage in ironing and folding on an average day, spending on average 48 minutes on this task. Of men, 3% engage in ironing and folding, spending 31 minutes on it.

**7 Gardening**

Gardening consists of things like mowing the lawn, trimming a hedge, planting plants and vegetables, pulling weeds, ploughing, watering the garden, harvesting, and tending to outdoor flowers.

Note: Tending to indoor plants is included in "Cleaning the home".

In the UK, 6% of women engage in gardening on an average day, spending on average 68 minutes on this task. Of men, 5% garden, spending 85 minutes on it.

**8** **Pet care**

Pet care consists of things like walking a dog, feeding and washing pets and other domestic animals, taking care of an aquarium, bee-keeping, and grooming a horse.

In the UK, 21% of women engage in pet care on an average day, spending on average 55 minutes on this task. Of men, 16% engage in pet care, spending 61 minutes on it.

**9 Household and car maintenance**

Household and car maintenance consists of things like assembling furniture, repairing furniture and household equipment, checking the boiler, changing car tyres, repairing a bicycle, and renovating the home.

In the UK, 33% of women engage in household and car maintenance on an average day, spending on average 29 minutes on this task. Of men, 34% engage in houshold and car maintenance, spending 45 minutes on it.

**10 Grocery shopping**

Grocery shopping consists of things like shopping for groceries at supermarkets and local shops, ordering groceries online, and buying snacks from a kiosk.

Note: Includes both offline and online shopping. If you think that in the future people will increasingly switch to online shopping and home delivery, that may reduce the time spent on shopping, which counts as automation for the purposes of this survey.

Note: Shopping for other items (eg. clothes) is a separate task and is not included in grocery shopping.

In the UK, 20% of women engage in grocery shopping on an average day, spending on average 40 minutes on this task. Of men, 13% do grocery shopping, spending 38 minutes on it.

**11 Shopping (other than groceries)**

Shopping (other than groceries) consists of things like buying clothes, household appliances, and furniture; buying a car; and circling round looking for things to buy.

Note: Includes both offline and online shopping. If you think that in the future people will increasingly switch to online shopping and home delivery, that may reduce the time spent on shopping, which counts as automation for the purposes of this survey.

Note: Shopping does not include the use of services (eg. hairdresser), which is a separate task.

In the UK, 48% of women engage in shopping (other than groceries) on an average day, spending on average 79 minutes on this task. Of men, 34% do shopping, spending 76 minutes on it.

**12** **Using services**

Using services consists of things like visiting a doctor, dentist, hairdresser, or shoe repair shop; visiting a bank or post office; using an ATM to pay bills or withdraw cash; using online banking; calling customer support; and doing paperwork for government authorities.

**13 Physical childcare**

Physical childcare consists of things like feeding a child, supervising a child, and taking care of a sick child.

Note: Does not include interacting with a child, teaching a child, and escorting a child outside the home, which are separate tasks.

In the UK, 34% of women engage in physical childcare on an average day, spending on average 91 minutes on this task. Of men, 17% engage in physical childcare, spending 60 minutes on it.

**14 Teaching a child**

Teaching a child consists of things like helping a child with homework and guiding a child in doing things.

Note: Does not include physical childcare, interacting with a child, and escorting a child outside the home, which are separate tasks.

In the UK, 4% of women engage in teaching a child on an average day, spending on average 43 minutes on this task. Of men, 1% teach a child, spending 42 minutes on it.

**15** **Interacting with a child**

Interacting with a child consists of things like talking to a child, reading to a child, and playing with a child.

Note: Does not include physical childcare, teaching a child, and escorting a child outside the home, which are separate tasks.

In the UK, 20% of women engage in interacting with a child on an average day, spending on average 62 minutes on this task. Of men, 12% interact with a child, spending 65 minutes on it.

**16 Escorting a child outside the home**

Escorting a child outside the home consists of things like taking a child to school or nursery, taking a child to hobbies and waiting outside, and accompanying a child to a doctor's appointment.

Note: Does not include physical childcare, interacting with a child, and teaching a child, which are separate tasks.

In the UK, 18% of women engage in escorting a child outside the home on an average day, spending on average 63 minutes on this task. Of men, 8% escort a child outside the home, spending 56 minutes on it.

**17 Caring for an adult**

Caring for an adult consists of things like physical care and supervision for an elderly, sick, or disabled adult; accompanying an adult to a hospital; and providing mental help, information and advice to an adult.

In the UK, 8% of women engage in caring for an adult on an average day, spending on average 53 minutes on this task. Of men, 7% care for an adult, spending 47 minutes on it.

*After the completion of the questionnaire, we grouped the 17 tasks into two categories, “care task” and “non-care tasks” to differentiate between care work and house work. Task 1 to 12 are grouped as “care task”, and task 13 – 17 are grouped as “non-care task”.*

| **caretask** | **Care task** |
| --- | --- |
| 0 | Non-care task |
| 1 | Care task |

*After each task description, the following questions were shown. These variables show respondents’ answers in the 2^nd^ delphi survey wave:*

| **auto_5y** | **5 years from now, what percentage of the time that currently goes into this task can be automated?** |
| --- | --- |
| range [0,100] | Answer options are a numerical sliding scale ranging from 0-100 |

| **cost_5y** | **5 years from now, how much will it cost for a household to use this automation for 1 year?**  *Tip: For example, use your best guess of how such a solution would be marketed: eg., as a mass-market subscription service (£10/month = £120/year), or as a high-end household appliance (£1,500 purchase price, 3-year replacement cycle = £500/year). Another option is to use your best guess of production costs and assume that the solution is priced at cost.* |
| --- | --- |
| 1 | £0-10 |
| 2 | £11-100 |
| 3 | £101-1000 |
| 4 | £1001-10,000 |
| 5 | More than £10,000 |

| **auto_10y** | **10 years from now, what percentage of the time that currently goes into this task can be automated?** |
| --- | --- |
| range [0,100] | Answer options are a numerical sliding scale ranging from 0-100 |

| **cost_10y** | **10 years from now, how much will it cost for a household to use this automation for 1 year?** |
| --- | --- |
| 1 | £0-10 |
| 2 | £11-100 |
| 3 | £101-1000 |
| 4 | £1001-10,000 |
| 5 | More than £10,000 |

*The data set also includes respondents’ answers from the first delphi survey wave to the same questions. These variables have the prefix “w1_”:*

| **w1_auto_5y** | **5 years from now, what percentage of the time that currently goes into this task can be automated?** |
| --- | --- |
| range [0,100] | Answer options are a numerical sliding scale ranging from 0-100 |

| **w1_cost_5y** | **5 years from now, how much will it cost for a household to use this automation for 1 year?**  *Tip: For example, use your best guess of how such a solution would be marketed: eg., as a mass-market subscription service (£10/month = £120/year), or as a high-end household appliance (£1,500 purchase price, 3-year replacement cycle = £500/year). Another option is to use your best guess of production costs and assume that the solution is priced at cost.* |
| --- | --- |
| 1 | £0-10 |
| 2 | £11-100 |
| 3 | £101-1000 |
| 4 | £1001-10,000 |
| 5 | More than £10,000 |

| **w1_auto_10y** | **10 years from now, what percentage of the time that currently goes into this task can be automated?** |
| --- | --- |
| range [0,100] | Answer options are a numerical sliding scale ranging from 0-100 |

| **w1_cost_10y** | **10 years from now, how much will it cost for a household to use this automation for 1 year?** |
| --- | --- |
| 1 | £0-10 |
| 2 | £11-100 |
| 3 | £101-1000 |
| 4 | £1001-10,000 |
| 5 | More than £10,000 |

*In the last section of the questionnaire, we ask three questions about the respondent’s background*

| **gender** | **Gender** |
| --- | --- |
| 1 | Female |
| 2 | Male |

| **age** | Age |
| --- | --- |
| 1 | 20-24 |
| 2 | 25-29 |
| 3 | 30-34 |
| 4 | 35-39 |
| 5 | 40-44 |
| 6 | 45-49 |
| 7 | 50-54 |
| 8 | 55-59 |
| 9 | 60-64 |
| 10 | 65-69 |
| 11 | 70-74 |

| **expfield** | **Field of expertise (research team definition)** |
| --- | --- |
| 1 | Academia |
| 2 | R&D |
| 3 | Business |
